# CircCTNNA1 acts as a ceRNA for miR-363-3p to facilitate the progression of colorectal cancer by promoting CXCL5 expression

**DOI:** 10.1186/s40709-021-00135-8

**Published:** 2021-02-27

**Authors:** Yan Zhang, Sheng Zheng, Nansheng Liao, Huifeng Huang, Wenxiao Chen, Zhenxing Wu, Deqing Wu

**Affiliations:** 1 Department of Gastroenterology, Taizhou First People’s Hospital, Taizhou, 318020 China; 2 Department of Gastroenterology, The Third People’s Hospital of Yunnan Province, Kunming, 650011 China; 3 Department of General Surgery, Taizhou First People’s Hospital, Taizhou, 318020 China; 4 Department of Gastrointestinal Surgery, Guangdong Academy of Medical Sciences, School of Medicine, Guangdong Provincial People’s Hospital, South China University of Technology, No. 106 Zhongshan 2nd Road, Yuexiu District, Guangzhou, 510000 China

**Keywords:** Colorectal cancer, circCTNNA1, miR-363-3p, CXCL5

## Abstract

**Background:**

Circular RNAs (circRNA) have been shown to be involved in the pathogenesis of colorectal cancer (CRC). CircCTNNA1 was found to be one of the upregulated circRNAs in CRC. However, there are few studies on circCTNNA1, so it is necessary to carry out further studies.

**Methods:**

The expression of circCTNNA1, microRNA (miR)-363-3p, and chemokine C-X-C motif ligand 5 (CXCL5) was detected by quantitative real-time PCR (qRT-PCR). The protein levels of CXCL5 and metastasis markers were measured using western blot (WB) analysis. Cell proliferation, apoptosis, cell cycle, migration, and invasion were determined by cell counting kit 8 (CCK8) assay, colony formation assay, flow cytometry, and transwell assay. The relationship between miR-363-3p and circCTNNA1 or CXCL5 was evaluated via dual-luciferase reporter assay and RNA immunoprecipitation assay. Animal study was performed to explore the function of circCTNNA1 on CRC tumorigenesis.

**Results:**

CircCTNNA1 and CXCL5 were highly expressed in CRC. Knockdown of circCTNNA1 could inhibit the proliferation, cell cycle, metastasis, and promote the apoptosis of CRC cells. MiR-363-3p could be sponged by circCTNNA1, and the inhibition effect of circCTNNA1 silencing on CRC progression could be reversed by miR-363-3p inhibitor. Moreover, miR-363-3p could interact with CXCL5, and CXCL5 overexpression also could reverse the suppressive effect of miR-363-3p on CRC progression. Downregulation of circCTNNA1 also could hinder the tumor growth of CRC in vivo.

**Conclusion:**

CircCTNNA1 enhanced CRC progression via regulating the miR-363-3p/CXCL5 axis.

## Background

Colorectal cancer (CRC) is a common malignant tumor, often accompanied by distant metastasis of one or more organs [1]. In recent years, the incidence and mortality of CRC have shown an obvious upward trend in the world [2, 3]. Most CRC patients are in the middle and advanced stages when they are first diagnosed. Therefore, the detection of CRC biomarkers can help clinically formulate appropriate treatment plans [4, 5]. At present, the application of targeted therapy has brought the treatment of advanced CRC into a new stage [6, 7].

Circular RNA (circRNA) is an endogenous RNA molecule that can regulate gene expression, and it is widely present in eukaryotic cells [8, 9]. More and more circRNAs have been confirmed to participate in a variety of biological functions, which in turn affect the occurrence and development of diseases including cancers [10, 11]. Sufficient research confirms that the differential expression of circRNA is related to the malignant progression of cancer, which may be a potential target for cancer treatment [12, 13]. In CRC, many circRNAs have been found to participate in the regulation of CRC proliferation, metastasis and tumor generation, such as circ_103809 [14], circ_100290 [15] and circ-ZNF609 [16]. Circ_0074169 (derived from CTNNA1 gene, also called circCTNNA1) is a newly discovered circRNA in recent years. In the previous study, Chen et al*.* found that circCTNNA1 was an upregulated circRNA in CRC tissues, which could accelerate CRC proliferation and metastasis [17]. Therefore, circCTNNA1 might be a potential target of CRC. However, the research data of circCTNNA1 in CRC is still very limited, and its role and molecular mechanism in CRC are worthy of further exploration.

Chemokine C-X-C motif ligand 5 (CXCL5) belongs to the CXC family, which can bind to the G protein coupled receptor CXCR2, thereby affecting tumor growth, proliferation and metastasis [18, 19]. CXCL5 has been found to be significantly overexpressed in many cancers, including CRC, and has been shown to be an important oncogenic factor in cancer progression [20–22]. Interestingly, in our research, we discovered that circCTNNA1 and CXCL5 expression had a positively correlation in CRC, but it is unclear whether there was a regulatory relationship between the two.

Numerous studies have suggested that circRNA can be used as a sponge for microRNA (miRNA), by exerting the mechanism of competing endogenous RNA (ceRNA) to release the inhibition of miRNA on downstream genes, thereby indirectly regulating gene expression [23, 24]. Therefore, our research purpose is mainly to explore the role of circCTNNA1 in the progression of CRC. In addition, searching for the miRNA that interacts with circCTNNA1 and CXCL5 can reveal a new mechanism for circCTNNA1 regulating CRC progression.

## Methods

### Patient samples

Thirty seven paired CRC tumor tissues and adjacent normal tissues were collected from 37 CRC patients who had undergone surgery in Taizhou First People’s Hospital. Clinical tissue samples were frozen in liquid nitrogen.

### Cell culture

Human CRC cell lines (CACO2, LOVO, HT29, SW620 and SW480) were obtained from ATCC (Manassas, VA, USA) and normal colon mucosal epithelial cell line (NCM460) was purchased from Biovector NTCC Culture Center (Beijing, China). Cells were cultured in DMEM medium (Hyclone, South Logan, UT, USA) containing 10% fetal bovine serum (FBS, Hyclone) and 1% penicillin/streptomycin (Invitrogen, Carlsbad, CA, USA) at 37 °C with 5% CO_2_.

### Quantitative real-time PCR (qRT-PCR)

RNAiso Plus (Takara, Dalian, China) was used to extract total RNAs, and TIANscript RT Kit (Beijing, China) was used to obtain cDNA. According to the instructions of SYBR Premix Ex Taq II (Takara), qRT-PCR was conducted on PCR system. Relative expression was normalized by GAPDH or U6 and calculated using 2^−ΔΔCt^ method. The primer sequences were listed as below: circCTNNA1, F 5′-TGAGCCAGGAGTCTACACAGA-3′, R 5′-TGTTCACGGAAAACTTGGGCA-3′; CTNNA1, F 5′- GGGGATAAAATTGCGAAGGAGA-3′, R 5′-GTTGCCTCGCTTCACAGAAGA-3′; CXCL5, F 5′-AGCTGCGTTGCGTTTGTTTAC-3′, R 5′-TGGCGAACACTTGCAGATTAC-3′; miR-363-3p, F 5′-GCCGAGAATTGCACGGTAT-3′, R 5′-CTCAACTGGTGTCGTGGA-3′; GAPDH, F 5′-GGAGCGAGATCCCTCCAAAAT-3′, R 5′-GGCTGTTGTCATACTTCTCATGG-3′; U6, F 5′-TCTTCGTCATCACATATACTAAAAT-3′, R 5′-CTCTTCACGAATTTTCGTGTCAT-3′.

### Stability and localization of circRNA

For RNase R assay, the RNAs (2 μg) extracted from SW620 and SW480 cells were treated with 6 U RNase R (Geneseed Biotech, Guangzhou, China). The RNA not incubated with RNase R was used as control. After that, the RNA levels of circCTNNA1 and GAPDH (representative linear RNA) were detected by qRT-PCR.

For subcellular localization assay, the cytoplasm and nuclear RNAs of SW620 and SW480 cells were extracted with PARIS Kit (Invitrogen). Afterward, the expression of circCTNNA1, GAPDH (cytoplasm control) and U6 (nuclear control) in the nuclear and cytoplasm RNAs was detected by qRT-PCR.

### Western blot (WB) analysis

Total protein was extracted by RIPA Lysis Buffer (Sangon Biotech, Shanghai, China) and quantified with BCA Protein Assay Kit (Sangon Biotech). The same amount of protein was isolated by 10% SDS-PAGE gel and transferred to PVDF membrane (Beyotime, Shanghai, China). Membranes were immersed in 5% nonfat milk and then incubated with primary antibodies, including CXCL5 (ab9802, 1:10,000), E-cadherin (ab40772, 1:40,000), N-cadherin (ab18203, 1:1000) and GAPDH (ab9485, 1:2500). After incubating with Goat Anti-Rabbit IgG H&L (HRP) (ab205718, 1:50,000), the protein signals on the membranes were analyzed using ECL luminescent solution (Meilunbio, Dalian, China). All antibodies were bought from Abcam (Cambridge, MA, USA).

### Cell transfection

CircCTNNA1 small interfering RNAs (siRNAs), overexpressing vector and short hairpin RNA (shRNA) (si-circCTNNA1#1/#2, circCTNNA1 and sh-circCTNNA1), miR-363-3p mimic and inhibitor (miR-363-3p and in-miR-363-3p), pcDNA CXCL5 overexpression plasmid (CXCL5), or their negative controls (si-con, vector, sh-con, miR-con, in-miR-con and pcDNA) were obtained from Genepharma (Shanghai, China). SW620 and SW480 cells were seeded into 24-well plates, and transfection was conducted using Lipofectamine 3000 reagent (Invitrogen) when the cells reached 50–60% confluences.

### Cell counting kit 8 (CCK8) assay

The principle of CCK8 assay is mainly: WST-8 contained in CCK8 solution can be reduced by dehydrogenase in cell mitochondria to a highly water-soluble yellow formazan dye, and then the cell viability can be evaluated by detecting the amount of formazan. Specific steps were as follows: Cells were seeded in 96-well plates and cultured to the adherent wall. CCK8 solution (10 μL, Vazyme, Nanjing, China) was added to the plates at 4 time points (0, 24, 48 and 72 h) and incubated for 4 h. The optical density (OD) value was measured at 450 nm to assess cell viability.

### Colony formation assay

One hundred and fifty cells were inoculated on a 6-well plate. After 2 weeks, the colonies were immobilized with Paraformaldehyde Fix Solution (Beyotime, Shanghai, China), and then stained with Crystal Violet Staining Solution (Beyotime). Finally, the colonies were photographed and counted under a microscope.

### Transwell assay

Transwell chamber (8 μm, 24-well; Millipore, Billerica, MA, USA) was used to detect cell migration and invasion. The difference between cell migration and invasion was that an additional Matrigel (Solarbio, Beijing, China) was pre-coated on the bottom of the upper chamber in cell invasion assay. CRC cells were seeded into the upper chamber with DMEM medium, and the lower chamber was added with DMEM medium containing FBS. After 24 h, the cells were fixed with Paraformaldehyde Fix Solution, and stained with Crystal Violet Staining Solution. Cell number was counted using a microscope (100×).

### Flow cytometry

The cells were seeded into 6-well plates. After culturing for 48 h, the cells were digested and cell lysates were collected. Annexin V-FITC/propidium iodide (PI) Apoptosis Detection Kit (Keygen Biotech, Nanjing, China) was used for cell apoptosis. In brief, the cell lysates were suspended with Binding Buffer, and then incubated with Annexin V-FITC and PI for 10 min. Cell cycle was analyzed using Cell Cycle Detection Kit (Wanleibio, Shenyang, China). Briefly, the cell lysates were fixed in 70% ethanol, and then incubated with RNase A and PI for 30 min. Finally, the cell apoptotic rate and cell cycle distribution were analyzed using a flow cytometer.

### Dual-luciferase reporter assay

The binding sites of miR-363-3p in circCTNNA1 or CXCL5 3′UTR were predicted by the Starbase tool or Targetscan tool. According to the binding sequences, we constructed the wide-type (WT) and mutate-type (MUT) vectors of circCTNNA1 and CXCL5 3′UTR using the pGL3-control vector (Promega, Madison, WI, USA). The vectors and miR-363-3p mimic or miR-con were co-transfected into SW620 and SW480 cells. After 48 h, relative luciferase activities (Firefly/Renilla) were determined by Dual-Luciferase Reporter Assay Kit (Promega).

### RNA immunoprecipitation assay

According to the instruments of Magna RNA immunoprecipitation Kit (Millipore), RNA immunoprecipitation assay was performed. Briefly, SW620 and SW480 cells were lysed in RIP lysis buffer. Then, the cells lysates were incubated with magnetic beads conjugated with Anti-Ago2 or Anti-IgG. Finally, the RNA bounded to the magnetic beads were purified, and the expression of circCTNNA1, miR-363-3p and CXCL5 was measured by qRT-PCR.

### Animal study

SW620 cells were transfected with sh-con or sh-circCTNNA1 for 48 h, and then harvested and diluted with PBS (Beyotime). BALB/c nude mice (male, n = 10) were obtained from Vital River (Beijing, China) and randomly divided into 2 groups (n = 5). The diluted cells (2 × 10^6^ per 100 μL) were subcutaneously injected into the back of the mice. Every weekly, tumor volumes were measured by detecting tumor length and width using vernier caliper. Four weeks later, the mice were euthanized and tumor weight was analyzed.

### Statistical analysis

All statistical data were expressed as mean ± standard deviation in 3 independent experiments. The differences were assessed using Student’s *t*-test or one-way analysis of variance followed by Tukey’s test on GraphPad Prism 7.0 software (GraphPad, La Jolla, CA, USA). *p* value less than 0.05 was considered as statistically significant. Pearson coefficient correlation was used for conducting correlation analysis.

## Results

### CircCTNNA1 and CXCL5 were upregulated in CRC tumor tissues and cells

**Fig. 1:**
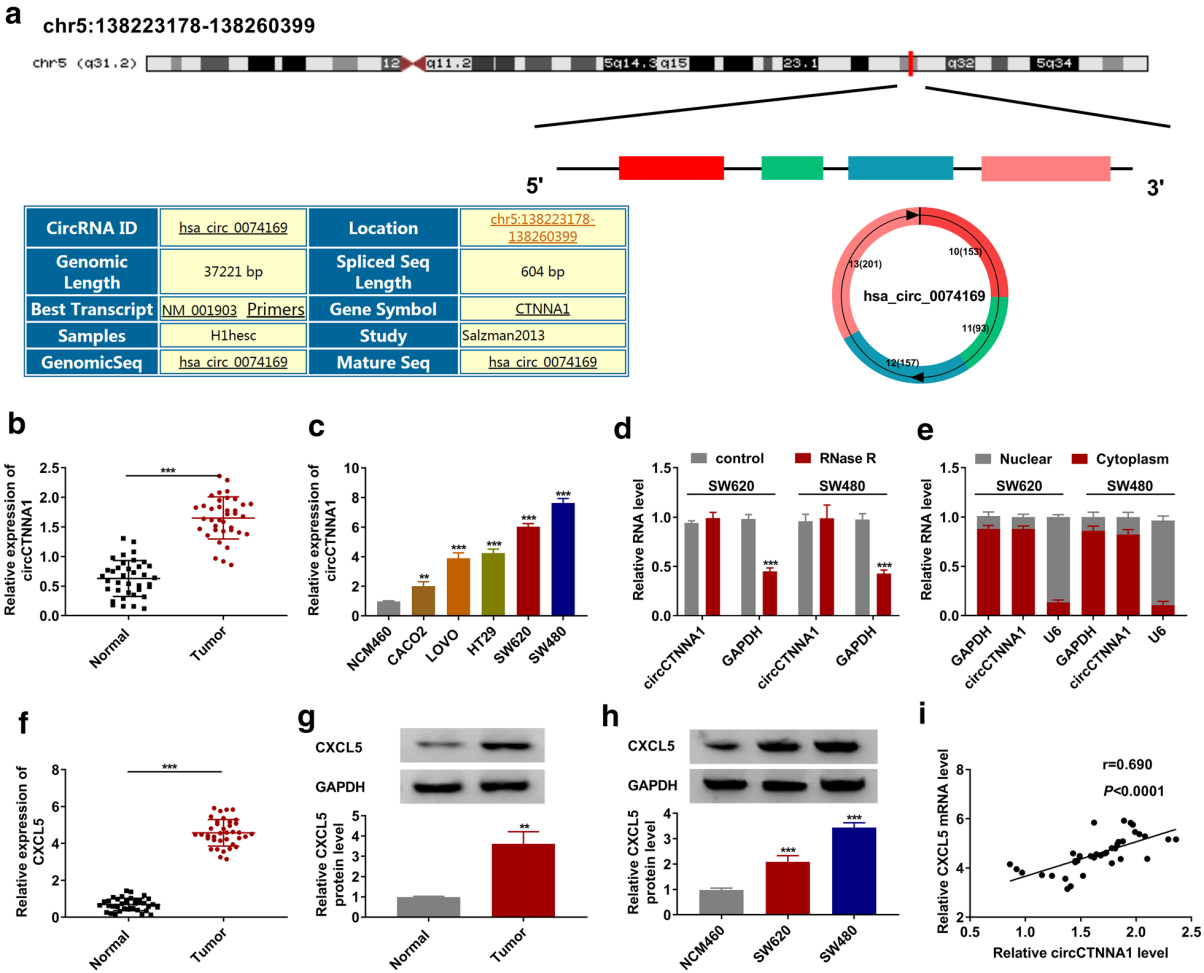
The expression of circCTNNA1 and CXCL5 in CRC tumor tissues and cells. **a** The diagram depicting the structure of the CTNNA1 gene and the structure of the circCTNNA1. **b** The expression of circCTNNA1 in CRC tumor tissues (Tumor) and adjacent normal tissues (Normal) was determined by qRT-PCR. **c** QRT-PCR was employed to detect the circCTNNA1 expression in NCM460 cells and CRC cell lines (CACO2, LOVO, HT29, SW620 and SW480). **d** RNase R assay was used to evaluate the stability of circCTNNA1 in SW620 and SW480 cells. **e** Subcellular localization assay was performed to confirm the distribution of circCTNNA1 in the nuclear and cytoplasm of SW620 and SW480 cells. **f**, **g** The mRNA and protein expression levels of CXCL5 in CRC tumor tissues (Tumor) and adjacent normal tissues (Normal) were examined using qRT-PCR and WB analysis. **h** WB analysis was used to test CXCL5 protein expression in NCM460 cells and both CRC cell lines (SW620 and SW480). **i** Pearson coefficient correlation was used to analyze the correlation between circCTNNA1 and CXCL5. **p < 0.01, ***p < 0.001

CircCTNNA1 is located on chr5:138,223,178–138,260,399 and is formed by cyclization of exon 10–13 of CTNNA1 gene with length of 604 bp (Fig. 1a). In the tumor tissues of CRC, we discovered that circCTNNA1 was highly expressed compared to the adjacent normal tissues (Fig. 1b). Moreover, circCTNNA1 expression also was obviously increased in five CRC cell lines, especially in SW620 and SW480 cells, compared with that in NCM460 cells (Fig. 1c). Further analysis showed that circCTNNA1 could effectively resist RNase R digestion compared with linear RNA GAPDH (Fig. 1d), and it was mainly distributed in the cytoplasm of CRC cells (Fig. 1e). This indicated that circCTNNA1 was a stable circRNA and might be mainly involved in post-transcriptional regulation. In addition, we also discovered that CXCL5 was markedly upregulated in CRC tumor tissues compared to adjacent normal tissues at the mRNA and protein levels (Fig. 1f, g). Also, we found a high expression of CXCL5 in SW620 and SW480 cells compared to NCM460 cells (Fig. 1h). Interestingly, correlation analysis showed a significant positive correlation between circCTNNA1 and CXCL5 expression in CRC tumor tissues (Fig. 1i).

### Silencing of circCTNNA1 suppressed proliferation, cell cycle, metastasis and enhanced apoptosis in CRC cells

**Fig. 2:**
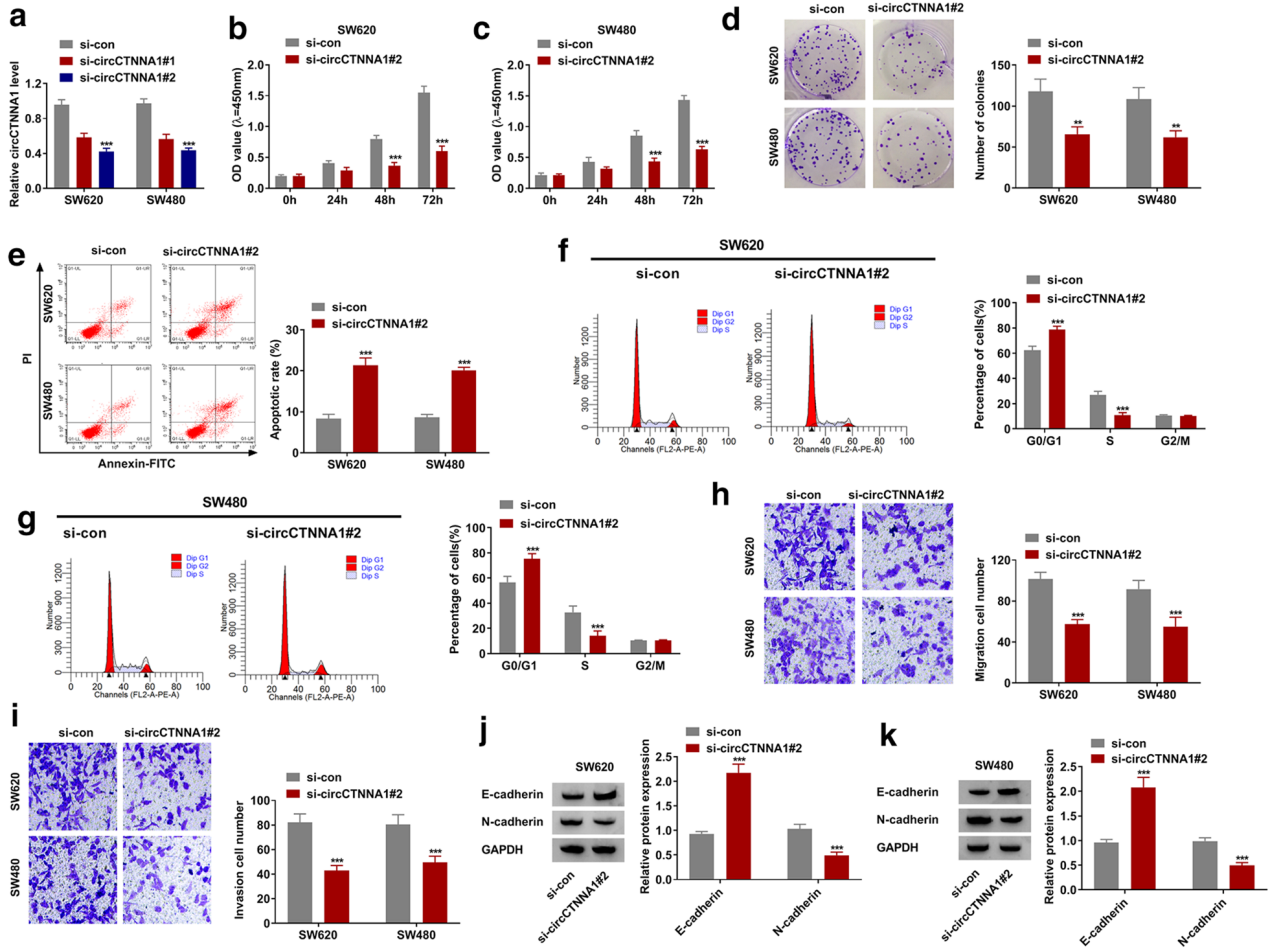
Silencing of circCTNNA1 suppressed CRC cell progression. **a** The transfection efficiencies of si-circCTNNA1#1 and si-circCTNNA1#2 were confirmed by detecting circCTNNA1 expression using qRT-PCR in SW620 and SW480 cells. **b**–**k** SW620 and SW480 cells were transfected with si-con or si-circCTNNA1#2. The viability, number of colonies, apoptotic rate, cell cycle, migration and invasion of cells were determined using CCK8 assay (**b**, **c**), colony formation assay (**d**), flow cytometry (**e**–**g**), and transwell assay (**h**, **i**). **j**, **k** The protein expression of E-cadherin and N-cadherin was measured using WB analysis. **p < 0.01, ***p < 0.001

To explore the effect of circCTNNA1 on CRC progression, the siRNA of circCTNNA1 was constructed and transfected into CRC cells. We found that both si-circCTNNA1#1 and si-circCTNNA1#2 significantly reduced circCTNNA1 expression in SW620 and SW480 cells (Fig. 2a), while had not effect on its linear RNA CTNNA1 expression (Additional file 1: Fig. S1A). The si-circCTNNA1#2 was used for functional testing because of its good inhibitory effect on circCTNNA1 expression. CCK8 assay suggested that silenced circCTNNA1 could inhibit the viability of SW620 and SW480 cells (Fig. 2b, c), and colony formation assay revealed that the number of colonies in SW620 and SW480 cells also could be reduced by circCTNNA1 knockdown (Fig. 2d). These evidences indicated that circCTNNA1 silencing could suppress the proliferation of CRC cells. Meanwhile, flow cytometry assay results showed that circCTNNA1 knockdown could enhance the apoptotic rate of SW620 and SW480 cells (Fig. 2e), and induce the cell cycle arrest in G0/G1 phase to reduce the cell number in S phase (Fig. 2f, g). Through transwell assay, we discovered that downregulated circCTNNA1 could significantly restrain the migration and invasion cell numbers in SW620 and SW480 cells (Fig. 2h, i). In addition, metastasis marker E-cadherin protein level was markedly increased, while N-cadherin protein level was notably decreased in the presence of circCTNNA1 silencing (Fig. 2j, k).

### CircCTNNA1 acted as a sponge for miR-363-3p

**Fig. 3:**
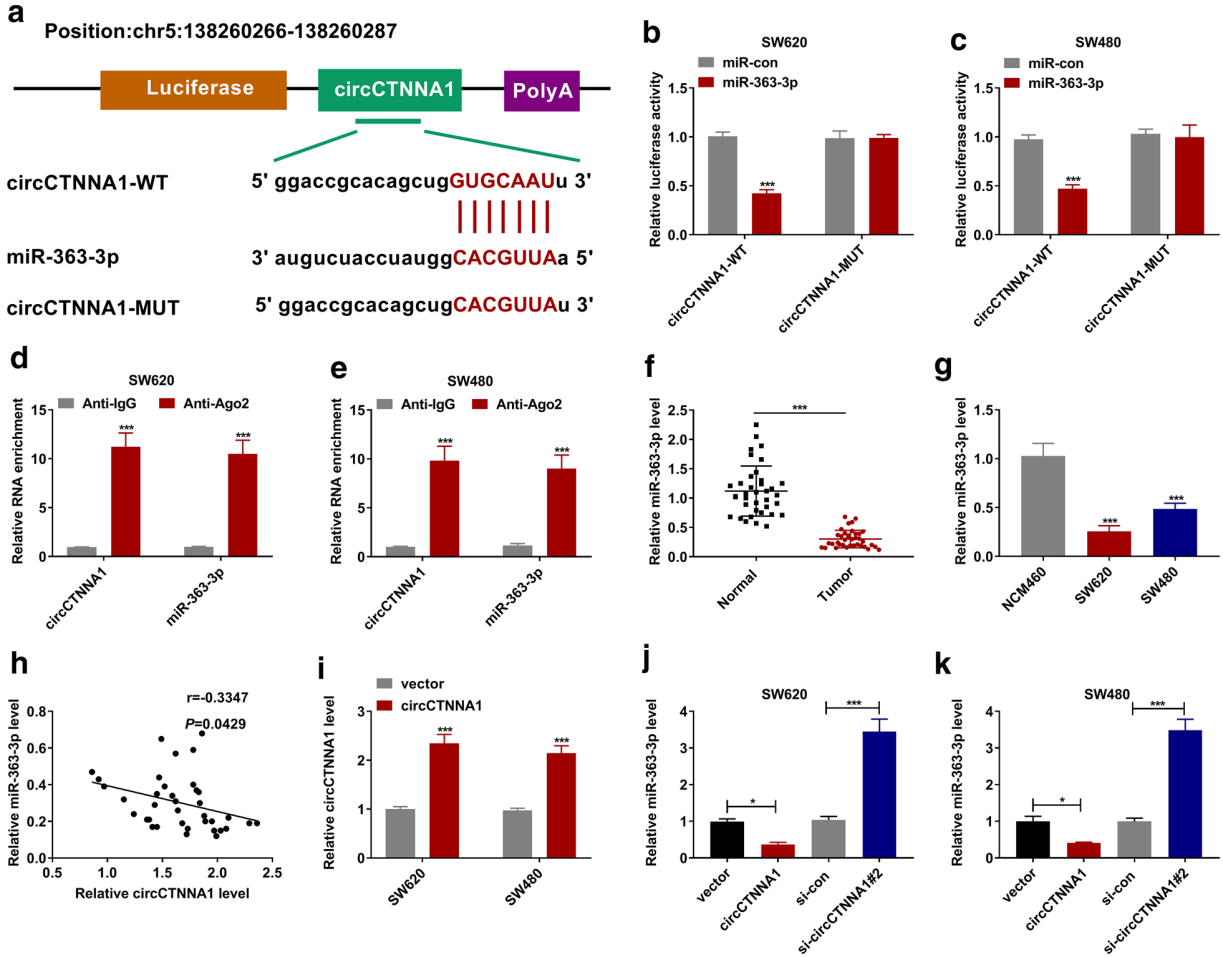
CircCTNNA1 acted as a sponged for miR-363-3p. **a** The putative binding sites of miR-363-3p in circCTNNA1 were presented. Dual-luciferase reporter assay (**b**, **c**) and RNA immunoprecipitation assay (**d**, **e**) were used to verify the interaction between miR-363-3p and circCTNNA1. **f** The expression of miR-363-3p in CRC tumor tissues (Tumor) and adjacent normal tissues (Normal) was tested by qRT-PCR. **g** QRT-PCR was used to measure the miR-363-3p expression in NCM460 cells and both CRC cell lines (SW620 and SW480). **h** The correlation between miR-363-3p and circCTNNA1 was evaluated using Pearson coefficient correlation. **i** The transfection efficiency of circCTNNA1 overexpressing vector was confirmed by testing circCTNNA1 expression in SW620 and SW480 cells. **j**, **k** SW620 and SW480 cells were transfected with vector, circCTNNA1, si-con or si-circCTNNA1#2. The expression of miR-363-3p was measured by qRT-PCR. *p < 0.05, ***p < 0.001

To find the miRNAs that interacted with circCTNNA1, bioinformatic analysis software (Starbase) was used for prediction. The results showed that miR-363-3p had a complementary binding site for circCTNNA1. Based on the binding sequences, we constructed the vectors of circCTNNA1-WT and circCTNNA1-MUT as shown in Fig. 3a. Dual-luciferase reporter assay indicated that miR-363-3p overexpression could reduce the luciferase activity of the circCTNNA1-WT vector, but not effect on that of the circCTNNA1-MUT vector in SW620 and SW480 cells (Fig. 3b, c). Moreover, we also found that the enrichment of circCTNNA1 and miR-363-3p in Anti-Ago2 was significantly higher than that of in Anti-IgG through the RNA immunoprecipitation assay (Fig. 3d, e). In CRC tissues and cells, we found a significantly lower expression of miR-363-3p (Fig. 3f, g), and we also discovered that the expression of miR-363-3p was negatively correlated with circCTNNA1 expression in CRC tissues (Fig. 3h). To further investigate the regulation of circCTNNA1 on miR-363-3p, the circCTNNA1 overexpressing vector was constructed. As presented in Fig. 3i, the circCTNNA1 overexpressing vector could obviously enhance circCTNNA1 expression in SW620 and SW480 cells. By measuring miR-363-3p expression, we uncovered that circCTNNA1 overexpression could significantly inhibit miR-363-3p expression, while its knockdown had an opposite effect in SW620 and SW480 cells (Fig. 3j, k).

### MiR-363-3p inhibitor overturned the inhibition effect of circCTNNA1 silencing on CRC progression

**Fig. 4:**
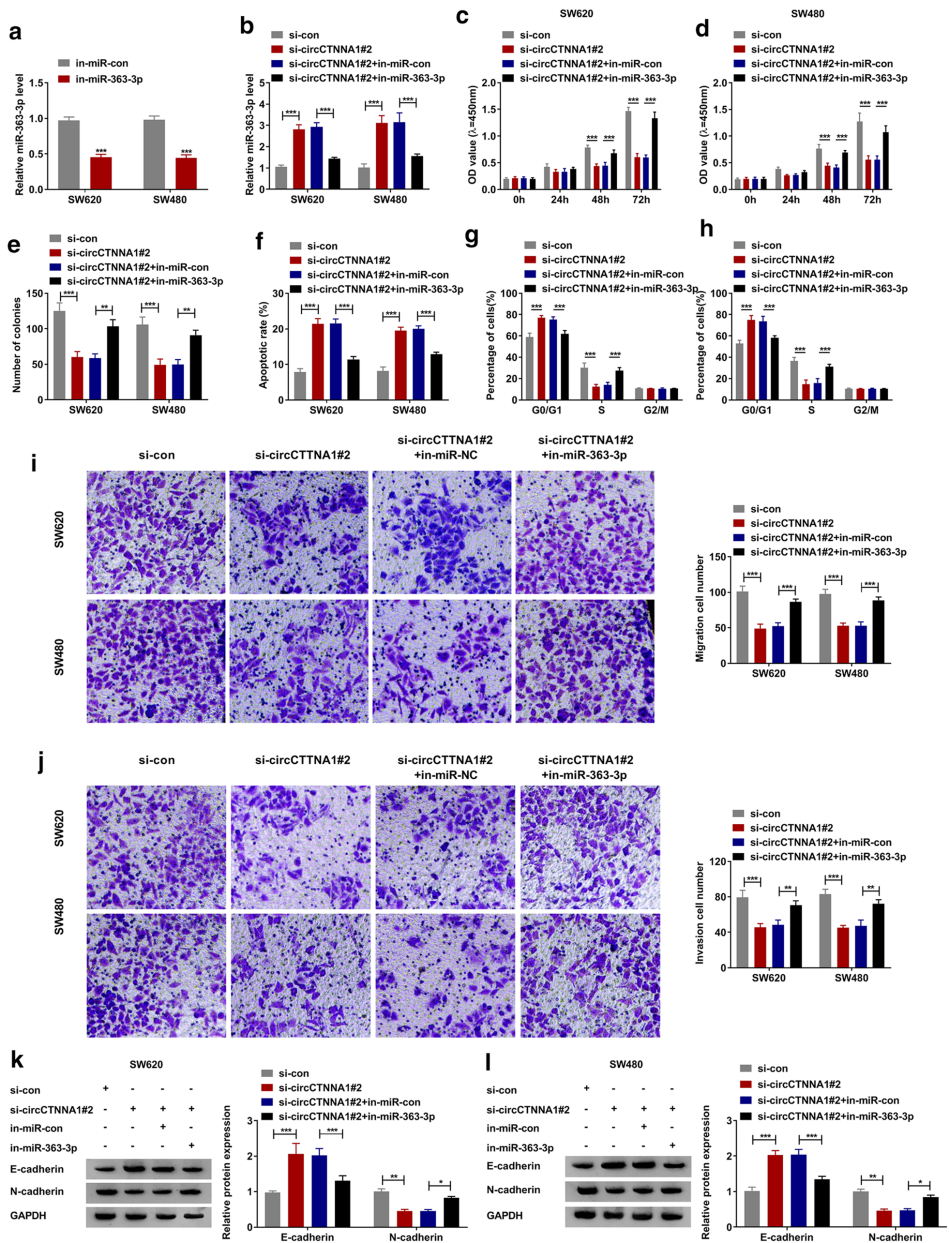
Effects of circCTNNA1 silencing and miR-363-3p inhibitor on CRC progression. **a** The transfection efficiency of in-miR-363-3p was verified by detecting miR-363-3p expression using qRT-PCR in SW620 and SW480 cells. **b**–**l** SW620 and SW480 cells were transfected with si-con, si-circCTNNA1#2, si-circCTNNA1#2 + in-miR-con or si-circCTNNA1#2 + in-miR-363-3p. **b** QRT-PCR was used to measure miR-363-3p expression. CCK8 assay (**c**, **d**), colony formation assay (**e**), flow cytometry (**f**–**h**), and transwell assay (**i**, **j**) were employed to determine the viability, number of colonies, apoptotic rate, cell cycle, migration and invasion of cells. **k**, **l** WB analysis was used to examine the protein expression of E-cadherin and N-cadherin. *p < 0.05, **p < 0.01, ***p < 0.001

To investigate whether circCTNNA1 regulated CRC progression by sponging miR-363-3p, in-miR-363-3p was built and its transfection efficiency was confirmed by detecting miR-363-3p expression in SW620 and SW480 cells transfected with in-miR-363-3p (Fig. 4a). Then, si-circCTNNA1#2 and in-miR-363-3p were co-transfected into SW620 and SW480 cells. As shown in Fig. 4b, the inhibitory effect of si-circCTNNA1#2 on miR-363-3p expression could be effectively reversed by in-miR-363-3p, which further confirmed that the transfection of the two was effective. CCK8 assay and colony formation assay results revealed that the suppressive effect of circCTNNA1 silencing on the viability and the number of colonies in SW620 and SW480 cells could be reversed by miR-363-3p inhibitor (Fig. 4c–e). Furthermore, the promoting effect of circCTNNA1 knockdown on cell apoptotic rate and the blocking effect on cell cycle could also be recovered by adding miR-363-3p inhibitor (Fig. 4f–h). Additionally, the addition of miR-363-3p inhibitor also reversed the inhibition effect of circCTNNA1 silencing on the migration and invasion of SW620 and SW480 cells (Fig. 4i, j). The detection results of E-cadherin and N-cadherin protein levels revealed that the increasing effect of circCTNNA1 knockdown on E-cadherin protein level and the decreasing effect on N-cadherin protein level could also be reversed by miR-363-3p inhibitor (Fig. 4k, l).

### MiR-363-3p directly targeted CXCL5

**Fig. 5:**
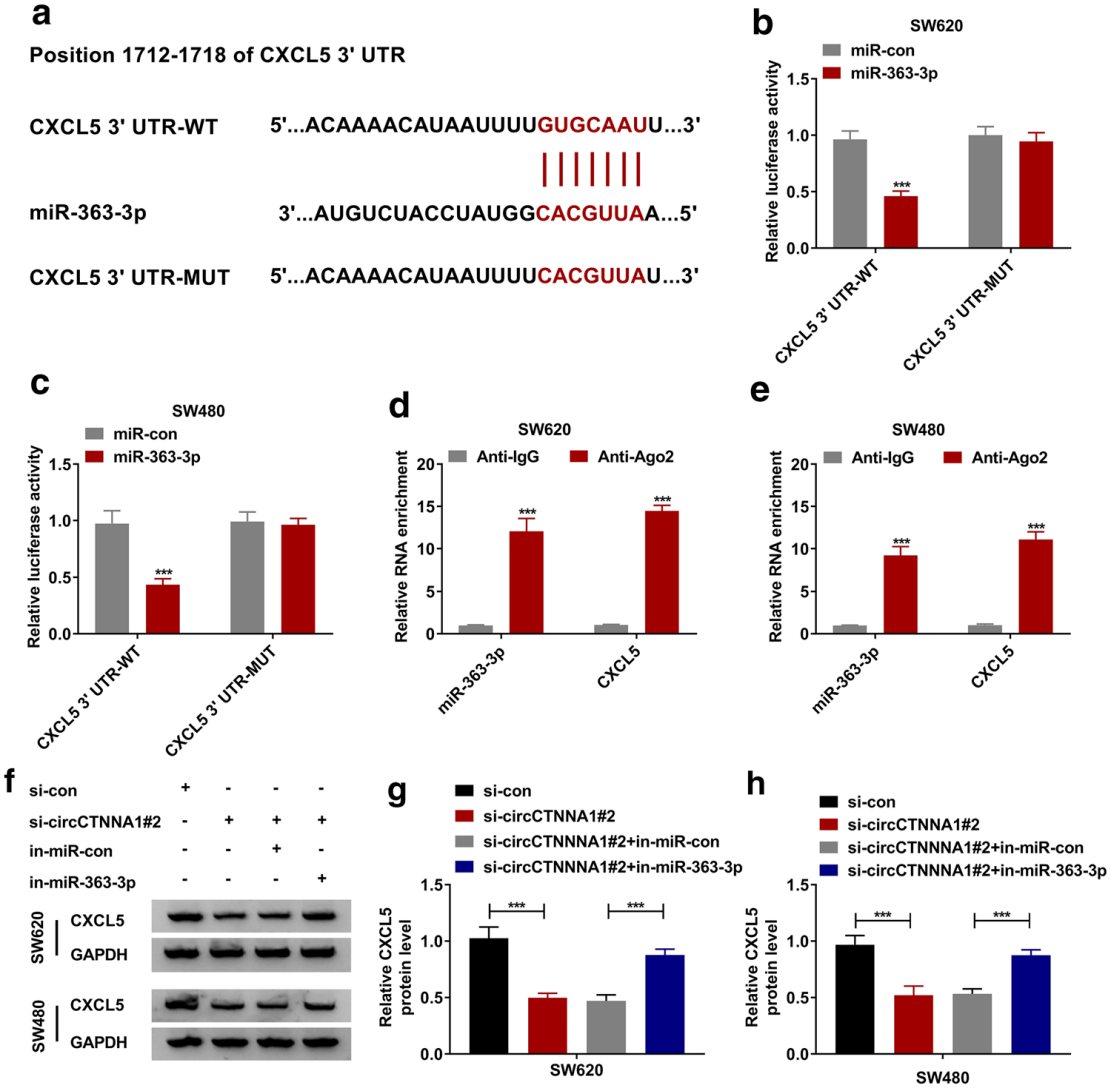
MiR-363-3p directly targeted CXCL5. **a** The binding sites between CXCL5 3′UTR and miR-363-3p were exhibited. The interaction between CXCL5 and miR-363-3p was confirmed by dual-luciferase reporter assay (**b**, **c**) and RNA immunoprecipitation assay (**d**, **e**). **f**–**h** SW620 and SW480 cells were transfected with si-con, si-circCTNNA1#2, si-circCTNNA1#2 + in-miR-con or si-circCTNNA1#2 + in-miR-363-3p. The protein expression of CXCL5 was detected by WB analysis. *** *p* < 0.001

Using the Targetscan online tool, we found that miR-363-3p and CXCL5 3′UTR had complementary binding sites (Fig. 5a). The dual-luciferase reporter assay results also showed that miR-363-3p could inhibit the luciferase activity of the CXCL5 3′UTR-WT vector without affecting that of the CXCL5 3′UTR-MUT vector (Fig. 5b, c). Further RNA immunoprecipitation assay confirmed that miR-363-3p and CXCL5 could be significantly enriched in Anti-Ago2 (Fig. 5d, e). These results indicated that CXCL5 was a target of miR-363-3p. To confirm the regulatory effects of circCTNNA1 and miR-363-3p on CXCL5, we detected CXCL5 protein level in SW620 and SW480 cells co-transfected with si-circCTNNA1#2 and in-miR-363-3p. The results showed that circCTNNA1 knockdown could inhibit CXCL5 expression, while this effect could be reversed by miR-363-3p inhibitor (Fig. 5f–h).

### The negative regulation of miR-363-3p on CRC progression could be reversed by CXCL5

**Fig. 6:**
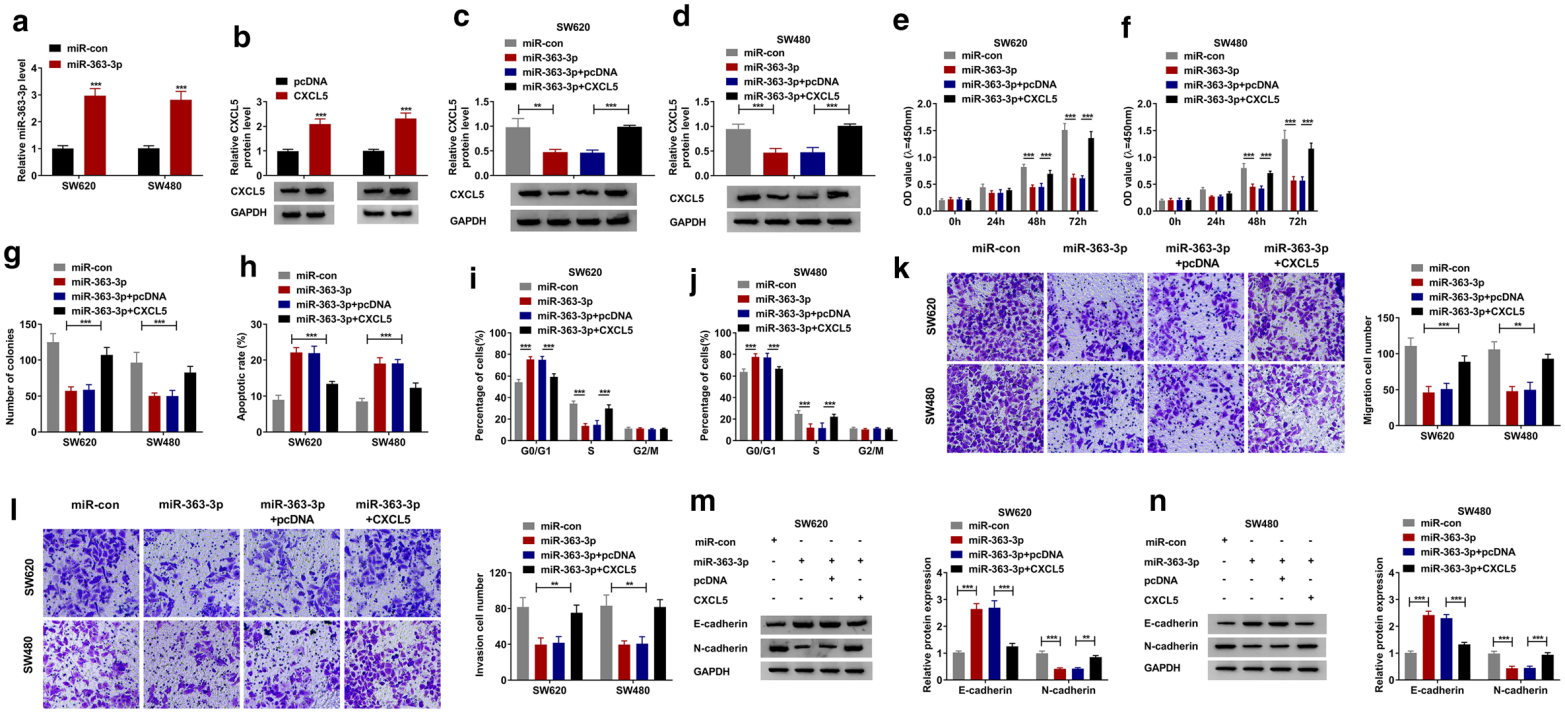
The negative regulation of miR-363-3p on CRC progression could be reversed by CXCL5. **a**, **b** The transfection efficiencies of miR-363-3p mimic and pcDNA CXCL5 overexpression plasmid were assessed by detecting miR-363-3p expression and CXCL5 protein expression in SW620 and SW480 cells using qRT-PCR and WB analysis, respectively. **c**–**n** SW620 and SW480 cells were transfected with miR-con, miR-363-3p, miR-363-3p + pcDNA or miR-363-3p + CXCL5. **c**, **d** The protein expression of CXCL5 was measured by WB analysis. CCK8 assay (**e**, **f**), colony formation assay (**g**), flow cytometry (**h**–**j**), and transwell assay (**k**, **l**) were used to examine the viability, number of colonies, apoptotic rate, cell cycle, migration and invasion of cells. **m**, **n** The protein expression levels of E-cadherin and N-cadherin were detected by WB analysis. *p < 0.05, **p < 0.01, ***p < 0.001

For determining that miR-363-3p regulated CRC progression by targeting CXCL5, the rescue experiments were carried out using miR-363-3p mimic and pcDNA CXCL5 overexpression plasmid. In the SW620 and SW480 cells transfected with miR-363-3p mimic or pcDNA CXCL5 overexpression plasmid, the expression levels of miR-363-3p and CXCL5 were significantly increased, indicating that the transfection efficiency of both was good (Fig. 6a, b). Subsequently, miR-363-3p mimic and pcDNA CXCL5 overexpression plasmid were co-transfected into SW620 and SW480 cells. By detecting CXCL5 protein level, we found that the inhibitory effect of miR-363-3p on CXCL5 level could be effectively restored by pcDNA CXCL5 overexpression plasmid (Fig. 6c, d). MiR-363-3p could inhibit the viability and the number of colonies, while promote the apoptosis and cell cycle arrest in SW620 and SW480 cells. However, these effects could be reversed by CXCL5 overexpression (Fig. 6e–j). Similarly, the suppressive effects of miR-363-3p on the migration and invasion of SW620 and SW480 cells also were reversed by CXCL5 overexpression (Fig. 6k, l). Additionally, miR-363-3p also promoted the E-cadherin protein expression and reduced the N-cadherin protein expression, while CXCL5 overexpression reversed these effects (Fig. 6m, n).

### CircCTNNA1 knockdown inhibited CRC tumorigenesis in vivo

**Fig. 7:**
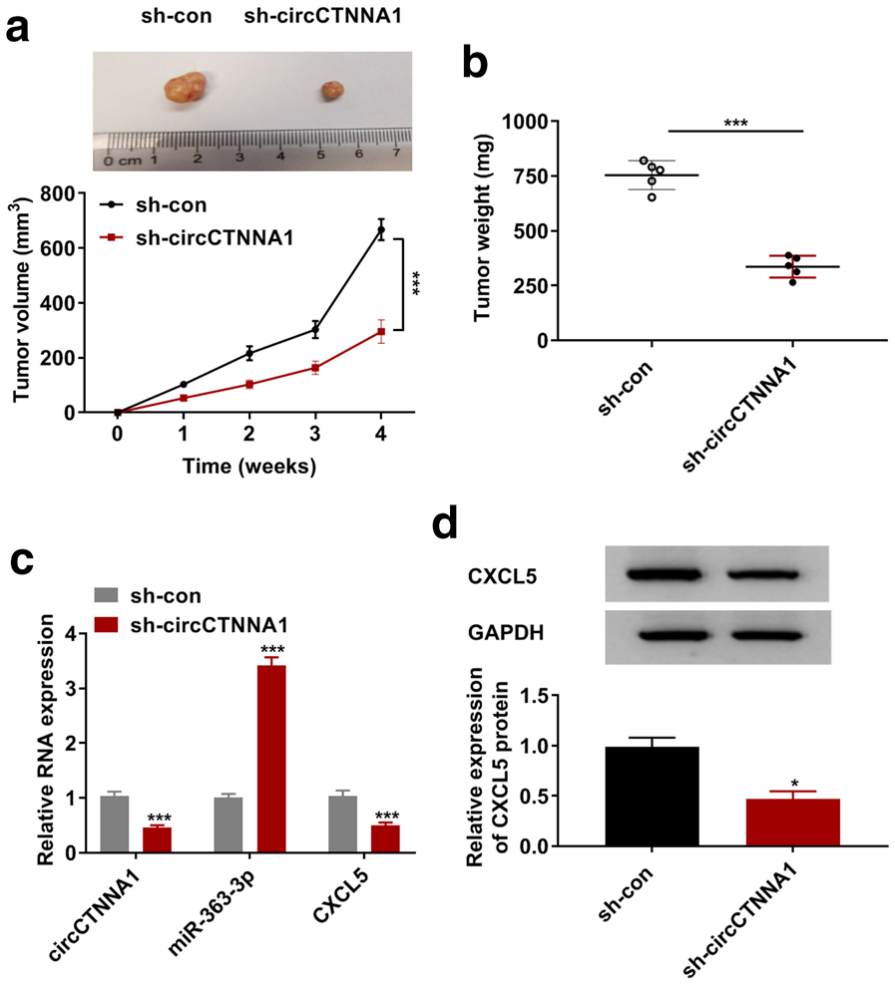
CircCTNNA1 knockdown inhibited CRC tumorigenesis in vivo*.* SW620 cells transfected with sh-con or sh-circCTNNA1 were injected into nude mice. Tumor volume (**a**) and tumor weight (**b**) in each group were determined. **c** The expression levels of circCTNNA1, miR-363-3p and CXCL5 in the tumor tissues of each group were detected using qRT-PCR. **d** The protein expression of CXCL5 in the tumor tissues of each group was measured using WB analysis. *p < 0.05, ***p < 0.001

In addition, we constructed the subcutaneous xenograft tumors to investigate the effect of circCTNNA1 silencing on CRC tumor growth. By subcutaneous injection of SW620 cells transfected with sh-circCTNNA1 or sh-con, we found that the volume and weight of subcutaneous xenograft tumors in mice in the circCTNNA1 knockdown group were significantly lower than those in the control group (Fig. 7a, b). In the tumors of the sh-circCTNNA1 group, we discovered that circCTNNA1 expression was significantly decreased and miR-363-3p expression was markedly increased compared with that in the control group (Fig. 7c). Moreover, the mRNA and protein expression levels of CXCL5 were obviously hindered in the sh-circCTNNA1 group compared to the sh-con group (Fig. 7c, d). In addition, linear RNA CTNNA1 expression had not any changed in the sh-circCTNNA1 group and sh-con group, confirming that the sh-circCTNNA1 did not affect the expression of CTNNA1 (Additional file 1: Fig. S1B).

## Discussion

Recently, although the treatment methods have improved, the prognosis of CRC patients has not yet reached the ideal. Therefore, the study of new diagnostic and therapeutic molecular markers is necessary to improve the prognosis of CRC patients. Due to its high stability, circRNA has been identified as an ideal biomarker for many diseases including cancer [12, 13]. With the continuous exploration of cancer-related circRNA, a large number of circRNA have been confirmed to be related to the tumorigenesis of CRC. Here, the upregulation of circCTNNA1 in CRC tissues and cells also was observed in our study. Further research found that circCTNNA1 expression was stable in CRC cells and mainly existed in the cytoplasm, which was consistent with the circular characteristics of circRNA.

Through silencing circCTNNA1 in CRC cells, we explored the circCTNNA1 role in CRC. Our results revealed that circCTNNA1 knockdown restrained the proliferation, cell cycle, migration and invasion of CRC cells, while enhanced cell apoptosis. In vivo experiments suggested that downregulated circCTNNA1 had a significant inhibitory effect on CRC tumorigenesis. Based on the above results, we believed that circCTNNA1 played a positive role in the progression of CRC, which was consistent with the results of previous studies [17]. In addition, we found a significant positive correlation between circCTNNA1 expression and CXCL5 expression in CRC tissues. This is an exciting discovery because CXCL5 is a well-known oncogenic factor. In CRC, CXCL5 had been found to promote tumor angiogenesis [25], and serum CXCL5 was considered to be an indicator of prognosis in CRC patients [26]. In particular, Zhao et al*.* demonstrated that CXCL5 could participate in the regulation of CRC progression as a promoter of CRC metastasis [22].

The ceRNA mechanism of circRNA has been confirmed by a large number of studies. Therefore, in order to clarify the potential relationship between circCTNNA1 and CXCL5, we conducted bioinformatic analysis to predict the miRNAs that could interact with circCTNNA1 and CXCL5. Combined with further experimental verification, we found that circCTNNA1 could serve as a ceRNA of miR-363-3p, and miR-363-3p could target CXCL5. MiR-363-3p is widely under-expressed in cancer and is considered as a tumor suppressor in many cancers, including renal cell carcinoma [27], lung adenocarcinoma [28], non-small-cell lung cancer [29], and papillary thyroid carcinoma [30]. In the previous studies, miR-363-3p was discovered to inhibit CRC proliferation and metastasis via targeting SphK2, EZH2 and Sox4 [31–33]. In our study, we also found a downregulated miR-363-3p in CRC tissues and cells. Functional experiments confirmed that circCTNNA1 regulated CRC progression via sponging miR-363-3p, and miR-363-3p targeted CXCL5 to inhibit CRC progression. In view of the positive regulation of circCTNNA1 on CXCL5 expression, we proposed that circCTNNA1 regulated the expression of the pro-oncogenic factor CXCL5 by targeting miR-363-3p.

## Conclusion

In conclusion, we proposed a new regulation network of circCTNNA1 in CRC. Our results confirmed that circCTNNA1 played a pro-cancer role in CRC, which could serve as the ceRNA of miR-363-3p to promote the CXCL5 expression. These results provide new evidence for circCTNNA1 as a biomarker of CRC.

## Supplementary Information

**Additional file 1: Fig. 1.** The effect of siRNA or shRNA of circCTNNA1 on CTNNA1 expression. (A) SW480 and SW620 cells were transfected with si-con, si-circCTNNA1#1 or si-circCTNNA1#2. The expression of CTNNA1 was measured by qRT-PCR. (B) SW620 cells transfected with sh-con or sh-circCTNNA1 were injected into nude mice. QRT-PCR was used to detect CTNNA1 expression in the tumor tissues of each group.

## Data Availability

The data sets used and/or analyzed during the current study are available from the corresponding author on reasonable request.
