# Supplementary figures and images for: CircCTNNA1 acts as a ceRNA for miR-363-3p to facilitate the progression of colorectal cancer by promoting CXCL5 expression
Source: J Biol Res (Thessalon). 2021 Feb 27;28:7. doi: 10.1186/s40709-021-00135-8 (PMC7913448; doi:10.1186/s40709-021-00135-8)

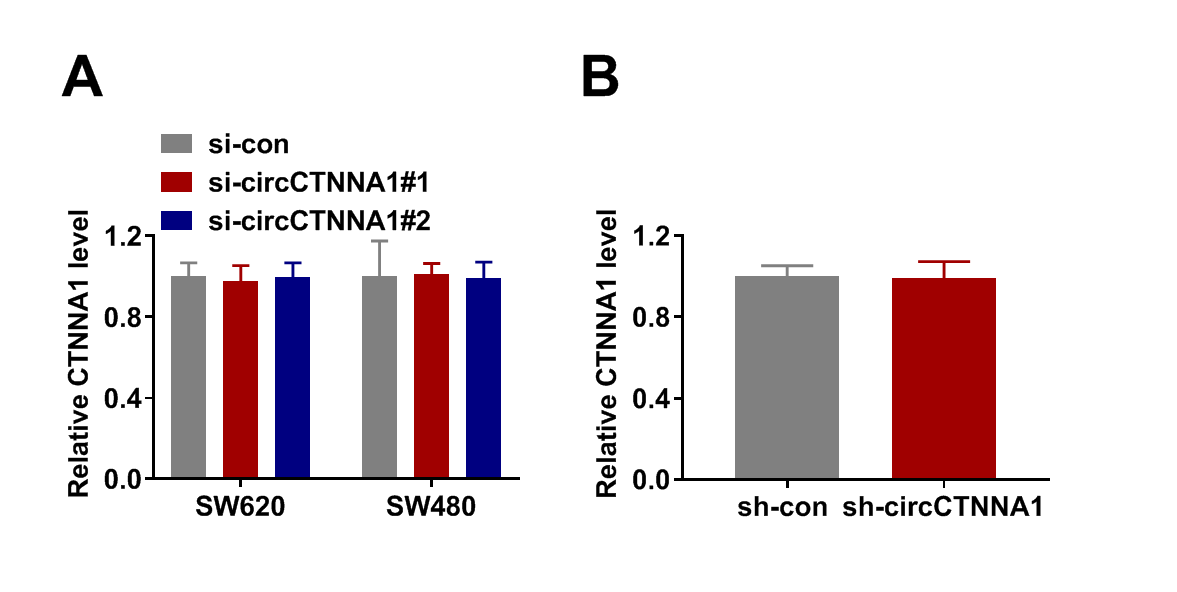

Supplement: Supplementary file 1 — Additional file 1: Fig. 1. The effect of siRNA or shRNA of circCTNNA1 on CTNNA1 expression. (A) SW480 and SW620 cells were transfected with si-con, si-circCTNNA1#1 or si-circCTNNA1#2. The expression of CTNNA1 was measured by qRT-PCR. (B) SW620 cells transfected with sh-con or sh-circCTNNA1 were injected into nude mice. QRT-PCR was used to detect CTNNA1 expression in the tumor tissues of each group. [file 40709_2021_135_MOESM1_ESM.tif]
